# The Gβ-like Protein *Af*CpcB Affects Sexual Development, Response to Oxidative Stress and Phagocytosis by Alveolar Macrophages in *Aspergillus fumigatus*

**DOI:** 10.3390/jof8010056

**Published:** 2022-01-06

**Authors:** Joo-Yeon Lim, Yeon-Ju Kim, Hee-Moon Park

**Affiliations:** 1 Institute of Biotechnology, Chungnam National University, 99 Daehak-ro, Yuseong-gu, Daejeon 34134, Korea; jooyeonlim1220@gmail.com; 2 Department of Microbiology and Molecular Biology, College of Bioscience and Biotechnology, Chungnam National University, Daejeon 34134, Korea; dw3623@naver.com

**Keywords:** *Aspergillus fumigatus*, CpcB, sexual development, alveolar macrophage, oxidative stress response, cell wall biosynthesis, ergosterol biosynthesis, hydrophobin, biofilm formation

## Abstract

G-protein signaling is important for signal transduction, allowing various stimuli that are external to a cell to affect its internal molecules. In *Aspergillus fumigatus*, the roles of Gβ-like protein CpcB on growth, asexual development, drug sensitivity, and virulence in a mouse model have been previously reported. To gain a deeper insight into *Aspergillus fumigatus* sexual development, the Δ*AfcpcB* strain was generated using the supermater AFB62 strain and crossed with AFIR928. This cross yields a decreased number of cleistothecia, including few ascospores. The sexual reproductive organ-specific transcriptional analysis using RNAs from the cleistothecia (sexual fruiting bodies) indicated that the CpcB is essential for the completion of sexual development by regulating the transcription of sexual genes, such as *veA, steA*, and *vosA*. The Δ*AfcpcB* strain revealed increased resistance to oxidative stress by regulating genes for catalase, peroxiredoxin, and ergosterol biosynthesis. The Δ*AfcpcB* strain showed decreased uptake by alveolar macrophages in vitro, decreased sensitivity to Congo red, decreased expression of cell wall genes, and increased expression of the hydrophobin genes. Taken together, these findings indicate that *Af*CpcB plays important roles in sexual development, phagocytosis by alveolar macrophages, biosynthesis of the cell wall, and oxidative stress response.

## 1. Introduction

*Aspergillus fumigatus* is found in indoor air, household dust, and outdoor environments, including soil and plant matter [1]. Fungal spores of *A. fumigatus* account for large proportions of air and can be inhaled deep into the lungs, resulting in health problems. Conidia are small enough (2–3 μm in diameter) to reach the human pulmonary alveoli [2,3]. The opportunistic fungal pathogen, *A. fumigatus*, is a leading agent of aspergillosis [4]. The inhaled *A. fumigatus* conidia can cause invasive aspergillosis in immunocompromised hosts [5]. The incidence of fungal infections has steadily increased and has become an emerging focus in recent decades [6].

The heterotrimeric G protein system is involved in spore germination, vegetative growth, stress response, and virulence in *Aspergillus nidulans* and *A. fumigatus* [7,8,9]. CpcB (cross-pathway control) is classified into Gβ-like proteins, which exhibit similar structures, including the WD-40 repeat motif, and forms dimers with the Gγ subunit [9]. While the c-Jun homolog CpcA inhibits sexual development under amino acid starvation, the RACK1 (receptor for activated protein kinase C) homolog CpcB is required for the sexual development in *A. nidulans* [10]. While deletion of *A. nidulans cpcB* (*AncpcB*) formed small and fragile cleistothecia that contain no ascospores [11], the function of the CpcB in *A. fumigatus* sexual development has not been investigated so far.

The sexual reproduction of *A. fumigatus* has been revealed to be induced on oatmeal agar under hypoxic and dark conditions in the laboratory [12]. Spherical white to yellow cleistothecia include ascus and ascospores in *A. fumigatus* [12]. The supermater AFB62 (*MAT1-1*) and AFIR928 (*MAT1-2*) strains, which produce abundant cleistothecia that contain viable ascospores, are suitable models for sexual development [13]. We have previously reported an improved sexual development-inducing method termed vegetative mass mating (VeM), in which mycelial balls—rather than conidia—from *MAT1-1* and *MAT1-2* strains were directly used on oatmeal agar [14]. While the VeM method results in the shortest production time (2 weeks) for full maturation of sexual reproductive organs and developmentally homogeneous cultures, this method is unable to achieve exclusive sexual development, as both asexual and sexual organs were observed [14].

Monocytes, macrophages, neutrophils, dendritic cells, and epithelial and endothelial cells, including other immune cells, contribute to the antifungal immune response [15]. LC3-associated phagocytosis (LAP) is a crucial noncanonical autophagy pathway that induces the killing of engulfed *A. fumigatus* conidia in monocytes and macrophages and is regulated by the NADPH oxidase complex, which is responsible for the production of antifungal reactive oxygen species (ROS) [16]. Pathogens have conserved structures recognized by pattern recognition receptors (PRR), termed pathogen-associated molecular patterns (PAMPs). Cell wall components of spores are major PAMPs, including chitin, glucan, and galactomannan [17]. *A. fumigatus* conidia express hydrophobin and melanin, which mask the cell wall component from PRRs of host cells and scavenge ROS against host defense mechanisms [18,19,20]. Hydrophobins belong to a unique small protein family (≤20 kDa) and are characterized by their hydrophobicity profiles and an idiosyncratic pattern of eight conserved cysteine residues [21]. Swelling or germination of conidia is accompanied by a loss of hydrophobin and melanin, which can cause an increase in the exposure of PAMPs to host immune cells [22]. Hypoxia has been observed in tissues and compartments during infection [23]. Immune cells, such as neutrophils, may adapt to a low-oxygen environment [24,25]. Fungal pathogens possess mechanisms to adapt to hypoxia and increased ROS in vivo during infection; sterol-regulatory element-binding protein has a role in hypoxia adaptation [25]. Hypoxia is involved not only in ROS production but also in the initiation of sexual development in *Aspergillus* spp. [13,26].

The families of antifungal drugs, such as echinocandins, polyene amphotericin B (AMB), and azoles are currently used for the treatment of aspergillosis, but treatment is hampered by the emergence of multi-azole-resistant isolates [27] and lack of oral formulations except for azoles, the only oral antifungals available [28]. Cyp51A, which is a 14α-demethylase involved in ergosterol biosynthesis, is a target of azoles [29]. Cryptic fungal species have different copies of *cyp51* proteins and can be inherently resistant [30]. Although many aspects of azole-resistance development in *A. fumigatus* have been studied, genetic diversity generated during the life cycle in fungi is crucial. Sexual crossing of azole-resistant *A. fumigatus* isolates shows the generation of resistance mutations, which suggests that fungi may acquire resistance mutations through sexual development [31]. Increased azole resistance is considered a global public health concern [32]. Sexual development can allow the organism to obtain drug resistance through recombination and random segregation of chromosomes. Therefore, it is necessary to conduct more studies on sexual development in *A. fumigatus*

In order to provide an adequate model for the role of *Af*CpcB in the sexual development of *A. fumigatus*, we constructed an *AfcpcB* deletion strain (*MAT1-1*) using the supermater AFB62 strain. Herein, we demonstrated that Gβ-like *Af*CpcB is involved in sexual development, phagocytosis by alveolar macrophages, biosynthesis of cell wall components, and response to external stressors.

## 2. Materials and Methods

### 2.1. The Strains, Media, and Growth Conditions

*A. fumigatus* AFB62 (*MAT1-1*) and AFIR928 (*MAT1-2*) strains, which represent a supermater pair, were obtained from the National Institutes of Health (NIH), USA. *A. fumigatus* strains were maintained in *Aspergillus* minimal medium with solid glucose (GMM) for 2–3 days [33]. For the production of the mycelial balls, YCMM (GMM with 0.15% yeast extract and 0.15% casamino acid) was used.

### 2.2. Generation of Strains

The *AfcpcB* deletion (∆*AfcpcB*) strain was constructed using the modified “Fusion PCR” method [34]. To construct disruption cassettes, the 5’ and 3’ regions of *cpcB* were amplified using PCR and *cpcB*-5’F/R and *cpcB*-3’F/R primer sets, respectively, along with the genomic DNA template. As a selection marker, a pyrithiamine (PT) resistance gene (*ptrA*) was amplified from plasmid pTRI by PCR using *ptrA*-F/R primers [35]. The three fragments were mixed and fused together by PCR using “nested” primers (*cpcB*-nestF/R). To construct the ∆*AfcpcB* strain, the disruption cassette was transformed into AFB62 protoplasts generated using VinoTaste^®^ Pro (Novozymes, Bagsvaerd, Denmark) [36]. Transformants were selected on GMM with pyrithiamine. To generate the *AfcpcB* complement strain, *hph* was amplified by PCR using pAN7-1 as a template and RF-TA-*hph*F/R primers. The PCR product was cloned into a pTA plasmid. The resulting plasmid pTH was digested with *Eco*RV and *Not*I. The 3’ regions of the *cpcB* gene were amplified using EZ *Eco*RV *cpcB* 3’F/EZ *cpcB* 3’ *Not*I R primers, along with the genomic DNA template, and cloned into the pTH. The resulting plasmid pTH3 was digested with *Hind*III and *Spe*I. The ORF and its predicted promoter region of the *cpcB* gene were amplified using EZ *Hind*III *cpcB* 5’F/EZ *cpcB* 5’ *Spe*I R primers and cloned into the pTH3 plasmid. The final amplicon was amplified with the *cpcB*-5’F/*cpcB*-nestR and introduced into a ∆*AfcpcB* strain. Transformants were selected on GMM with hygromycin and confirmed using PCR. Primers used in this study are shown in Supplementary Table S1.

### 2.3. Sensitivity Test to Chemical Agents

Spotting assays were performed as previously described [37]. Fresh conidia from 2-day-old cultures were collected and counted. Conidia (2 × 10^6^ /mL) were serially diluted 10-fold and 5 µL of each dilution was spotted onto a GMM containing 200 μg/mL of Congo red (CR) and 300 μg/mL of Calcofluor white (CFW) for cell wall stress, 10 μM menadione (MD) and 6 mM hydrogen peroxide (H_2_O_2_) for oxidative stress, and 1 μg/mL AMB for antifungal drug susceptibility. The plates were incubated for 2 days at 37 °C.

### 2.4. Physiological Studies

For sexual development in *A. fumigatus*, the VeM method was used [14]. Briefly, conidia of each *MAT1-1* or *MAT1-2* strain were inoculated in liquid YCMM and incubated at 37 °C. The mycelial balls cultured overnight were washed with distilled water and transferred onto solid oatmeal agar. The plate was sealed with parafilm and incubated at 30 °C for 2 weeks. For microscopic observation of the fungal sexual reproductive organs, cleistothecia were picked-up, rolled to detach the conidia, and ruptured on glass slides. The diameter of the cleistothecia was evaluated using ImageJ software (version 1.8.0, NIH).

### 2.5. Microscopy

For microscopy, an Olympus System differential interference contrast microscope model BX51 (Olympus, Tokyo, Japan) and the stereomicroscope SMZ800 (Nikon, Tokyo, Japan) were used. Images were captured using a DP71 digital camera (Olympus, Tokyo, Japan) and processed using the DP manager imaging software (Olympus, Tokyo, Japan).

### 2.6. RNA Isolation and Real-Time Quantitative PCR (RT-qPCR)

Mycelia were ground to powder using liquid nitrogen with a pestle and mortar [37]. Trizol (Invitrogen, Carlsbad, CA, USA) was used for total RNA extraction according to the manufacturer’s instructions. The total RNA in each cleistothecium was extracted using a modified version of Geoghegan’s method, which entails RNA extraction from the single conidium in *Aspergillus niger* [38]. Cleistothecium was obtained from 14-day mated cultures and rolled on a 2% agar plate to remove conidia around the cleistothecia. The cleistothecium was sorted into a centrifuge tube containing 100 μL of 0.5 mm zirconia/silica beads (BioSpec Product, Bartlesville, OK, USA), 300 μL of DEPC-treated water, and 1 μL of RNase inhibitor, and subsequently ground using a multimixer (EYELA, Tokyo, Japan) for 2 min. The aqueous phase was separated using centrifugation, transferred to a new tube, dried, and dissolved in 5 μL of DEPC-treated water. cDNA was synthesized from RNA using random hexamers (Enzynomics, Daejeon, Korea) and M-MLV reverse transcriptase (ELPIS biotech, Deajeon, Korea). Changes in SYBR green levels (Enzynomics, Deajeon, Korea) were measured using CFX96 Real-Time PCR (Bio-Rad Laboratories, Hercules, CA, USA). Relative fold changes in gene expression were determined using the 2^−ΔΔCT^ method [39]. Transcript levels of target genes were normalized against those of 18S rRNA [37].

### 2.7. Phagocytosis Assay

MH-S murine alveolar macrophages (CRL-2019) [40] were purchased from ATCC and cultured in RPMI medium containing 10% fetal bovine serum and 50 μM of 2-mercaptoethanol at 37 °C in an atmosphere of 5% CO_2_. A phagocytic assay was performed according to a modified method [41,42]. The MH-S cells were adhered to coverslips in 6-well plates at a concentration of 5 × 10^5^ cells/mL for 2 h and subsequently challenged with 1.5 × 10^6^ conidia (at a ratio of three conidia per macrophage) for 1 h. Unbound conidia were removed by washing with DPBS and then incubated for 2 h at 37 °C in an atmosphere of 5% CO_2_. Wells were then washed with DPBS and stained with CFW (1 μg/mL in DPBS) to label extracellular conidia [43]. Based on microscopic images, the percentage of phagocytosis and the phagocytosis index (the number of conidia per macrophage) were assessed.

### 2.8. β-glucan Analysis

The β-glucan amounts were determined using the enzymatic yeast beta-glucan Megazyme kit following the manufacturer’s protocol (Megazyme, Bray, Ireland).

### 2.9. Biofilm Formation Assay

Fungal biofilm assay was performed using 12-well plates as described previously [44]. Wells were inoculated with 1 mL of GMM media containing 10^5^ conidia and incubated for 16 h at 37 °C. Biofilms were washed three times with 3 mL of PBS and stained with 1 mL 0.01% (*w/v*) crystal violet solution and eluted with 30% acetic acid. Adhesion capacity was estimated by the absorbance at 550 nm using a spectrophotometer.

### 2.10. Statistical Analysis

Results are presented as the mean ± standard error of the mean (SEM) obtained from at least three independent experiments. Statistical differences were evaluated using Student’s *t*-test. Differences were considered significant at *p* < 0.05.

## 3. Results

### 3.1. Targeted Deletion of AfcpcB

**Figure 1:**
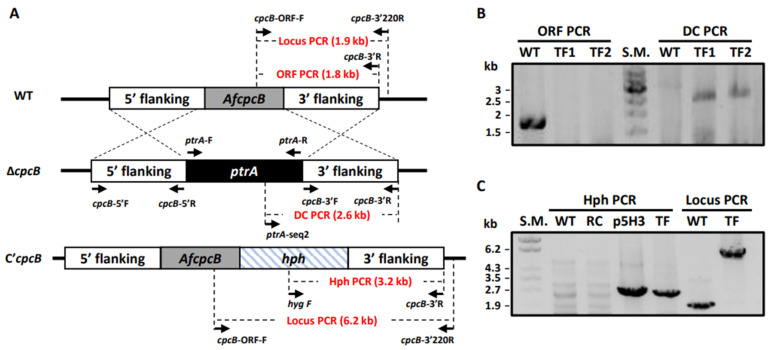
Generation of *A. fumigatus cpcB*-deletion and complementation stains. (**A**) Schematic illustration of the gene deletion and complementation method. (**B**) PCR confirmation of gene deletion. Genomic DNAs of WT, transformants 1 and 2 were extracted and used as a template for PCR reactions. Open reading frame (ORF) PCR products (1.8 kb) were amplified using *AfcpcB*-ORF-F and *cpcB*-3’R. Successful deletion was confirmed by 2.6 kb of disruption cassette (DC) PCR reaction using *ptrA*-seq2 and *cpcB*-3’R. Amplified PCR products were separated in a 0.8% agarose gel. (**C**) PCR confirmation of gene complementation. Hph PCR products (3.2 kb) were amplified using *hyg* F and *cpcB*-3’R. Locus PCR products (1.9 kb for WT and 6.2 kb for C’*cpcB* strain) were amplified using *cpcB*-ORF-F and *cpcB*-3’220R. TF, transformant; S.M., size marker; p5H3, plasmid carrying predicted promoter and ORF of *cpcB* and hygromycin resistance gene.

In *A. fumigatus*, *cpcB*-deletion strains were already generated in Af293.1 (*pyrG1*) and A1160 (Δ*ku80*; *pyrG*; *veA1*), termed Af293.1-7 and CZ01, respectively [9,11]. However, the Af293 strain is medium fertility, which produces low numbers of cleistothecia [13]. Ku70 and Ku80 form a heterodimer and play an important role in the non-homologous end joining (NHEJ) process in DNA repair [45]. The *akuB*^Ku80^ mutant showed an increased rate of homologous recombination, which resulted in the majority of transformed progenies with insertion mutagenesis [45,46]. While the Δ*ku80* strain is a useful tool to improve transformation efficiency, it is not suitable for the study of the sexual development required for the recombinational repair process [47]. To investigate the functional roles of *Af*CpcB in the sexual development in *A. fumigatus*, we generated the Δ*AfcpcB* strain in the supermater parental AFB62, which can produce a high number of cleistothecia [31]. The deletion construct was amplified using modified fusion PCR and employed for protoplast transformation. Putative transformants were grown on GMM agar containing pyrithiamine, and two candidates were selected based on PCR screening using *cpcB* ORF-F/*cpcB* 3’R and *ptrA*-seq2/*cpcB*-3’R primer sets for the open reading frame (ORF) PCR and disruption cassette (DC) PCR, respectively (Supplementary Table S1). As illustrated in Figure 1A, the genomic DNA of the transformants was not amplified using the ORF PCR primer set but amplified using the DC PCR primer set. Results from the PCR revealed a 1.8-kb band in the wild-type (WT) by ORF PCR and a 2.6-kb band in knock-out mutants based on DC PCR (Figure 1B). To complement the mutation, PCR fragments carrying a wild-type copy of *cpcB*, flanked by a hygromcyin resistance gene were used to transform Δ*AfcpcB* protoplasts. Complementation of the *cpcB* gene in the Δ*AfcpcB* strain background was confirmed by PCR using *hyg* F/*cpcB* 3’R and *cpcB-ORF-F*/*cpcB*-3’220R primer sets for Hph PCR and Locus PCR, respectively (Figure 1C).

### 3.2. AfCpcB Is Required for Complete Sexual Development

**Figure 2:**
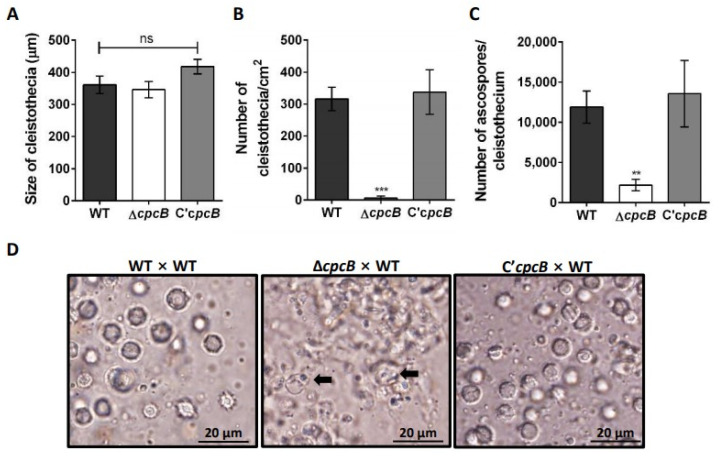
Effects of *Af*CpcB on sexual development. WT × WT pair (AFB62 and AFIR928 strains), Δ*cpcB* × WT pair (Δ*AfcpcB* and AFIR928 strains) and C’*AfcpcB* × WT pair (C’*AfcpcB* and AFIR928 strains) were used for sexual development induction by the vegetative mass mating method. (**A**) Size of cleistothecia. Diameter of randomly selected cleistothecia was measured using ImageJ software (*n* = 8). “ns” indicates not significantly different. (**B**) Quantification of the number of cleistothecia (*n* = 4). Statistical analysis was performed using Student’s *t*-test. *** *p* < 0.001. (**C**) Number of ascospores in a cleistothecium. Each cleistothecium was ruptured using 0.05% tween80 and counted using a hemocytometer (*n* = 8). Statistical analysis was performed using Student’s *t*-test. ** *p* < 0.01. (**D**) Differential interference contrast microscopic images of ruptured cleistothecia of 14-day-old cultures. Black arrow indicates irregular-shaped ascogenous cells.

To elucidate the role of *Af*CpcB in cleistothecia formation, the Δ*AfcpcB* strain (*MAT1-1*) was crossed with the supermater AFIR928 strain (*MAT1-2*) using the VeM method [13,14]. In oatmeal agar, *A. fumigatus* produces both asexual (green conidia) and sexual (white cleistothecia) reproductive organs [13]. Crossing the Δ*AfcpcB* strain with the AFIR928 strain (see Figure 2A, Δ*cpcB*) formed cleistothecia similar to the cleistothecia formed by crossing of the AFB62 with the AFIR928 strain (see Figure 2A, WT) and the C’*AfcpcB* strain with the AFIR928 strain (see Figure 2A, C’*cpcB*) (Figure 2A). However, the number of cleistothecia produced in the Δ*AfcpcB* × AFIR928 cross (6.3 ± 6.3 cleistothecia/cm^2^) was significantly lower than that in the WT pair (315.8 ± 36.4 cleistothecia/cm^2^) and C’*cpcB* pair (337.2 ± 69.6 cleistothecia/cm^2^) (Figure 2B). To investigate the formation of ascospores, which are final sexual reproductive organs, we picked up cleistothecia and ruptured them on glass slides. A cloud of ascospores with two equatorial crests was released from a cleistothecium formed by crossing the WT pair (Figure 2D, left) and the C’*AfcpcB* × WT pair (Figure 2D, right). In the Δ*AfcpcB* × AFIR928 cross, few or no ascospores were formed in cleistothecia. Instead, a mass of irregularly shaped ascogenous cells (black arrows) can be seen within the cleistothecia (Figure 2D, center). In agreement with phenotypic data, statistical analysis revealed that the cleistothecia in the Δ*AfcpcB* × AFIR928 cross contained only about 16~18% of the ascospores (2181 ± 706 ascospores) compared to that in the WT pair cross (11,891 ± 2006 ascospores) and the C’*AfcpcB* × WT pair (13,556 ± 4129 ascospores) (Figure 2C). Taken together, these data suggest that *Af*CpcB is required for the complete sexual development of *A. fumigatus*.

### 3.3. Transcript Expression Patterns in Cleistothecia

Mating induced by the VeM method on oatmeal agar produced a high number of both conidia and cleistothecia [14]. To investigate transcript expression patterns in only sexual reproductive organs containing ascus and ascospores, we performed the amplification of cDNA using total RNA extracted from cleistothecia.

**Figure 3:**
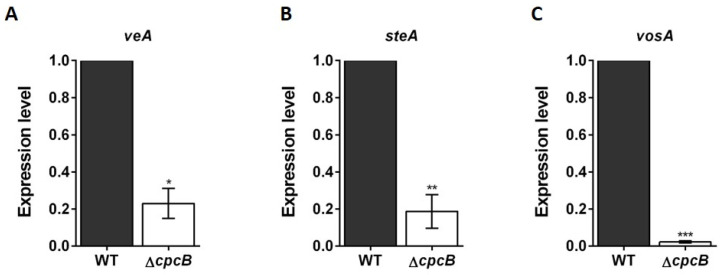
Expression pattern of the genes associated with sexual development, *veA* (**A**), *steA* (**B**), and *vosA* (**C**) genes, in cleistothecia (*n* = 4). In 14-day cultures containing both cleistothecia and conidia, a cleistothecium was collected and rolled onto a new agar plate using a stereomicroscope. The cleistothecium was transferred into a centrifuge tube with beads, RNase inhibitor, and DEPC-treated water and ground. Synthesized cDNA from this solution was used for RT-qPCR with primers binding to an exon–exon boundary. The RT-qPCR expression ratios were normalized using the 18S rRNA gene. Statistical analysis was performed using Student’s *t*-test. * *p* < 0.05, ** *p* < 0.01, *** *p* < 0.001.

To investigate the effect of *AfcpcB* deletion on the transcription levels of genes involved in sexual development, *veA*, *steA*, and *vosA* expression levels were detected in cleistothecia sorted from the AFB62 × AFIR928 and Δ*AfcpcB* × AFIR928 mated cultures. In *A. fumigatus*, VeA is required for conidiation, gliotoxin production, and protease activity [48]. In *A. nidulans*, VeA activates sexual development, and controlled gene expression is required during sexual development [11,37,49]. SteA (sterile 12-like) is a homeodomain-C_2_/H_2_-Zn^+2^ finger transcription factor, and the *steA*-deletion strain does not differentiate cleistothecia, ascogenous hyphae, asci, or ascospores, thereby suggesting that SteAp functions very early in the sexual cycle [50]. VosA (viability of spores) is required for spore viability, and the *vosA*-deletion strain produces defective cleistothecia containing a very small number of ascospores [51]. The *steA* and *vosA* genes are expressed in the early and late sexual stages, respectively [37]. When the transcription of sexual development-associated genes *veA*, *steA*, and *vosA* were analyzed in *A. fumigatus*, transcript levels of *veA*, *steA*, *vosA* genes were found to be significantly decreased by 4.3-fold, 5.3-fold, and 44.4-fold, respectively, in the cleistothecia that originated from the Δ*AfcpcB* × AFIR928 cross (Figure 3). These results indicate that *Af*CpcB is essential for the completion of sexual development by regulating the transcription of genes associated with early and late sexual development in *A. fumigatus*.

### 3.4. The ΔAfcpcB Mutant Is More Resistant to Phagocytosis by Alveolar Macrophages

**Figure 4:**
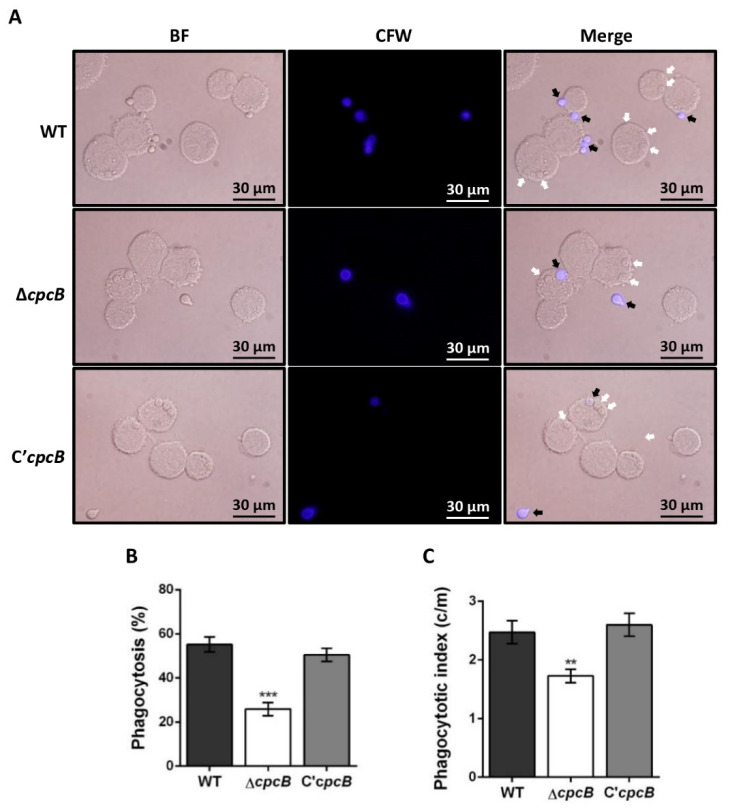
Alveolar macrophage response to WT, Δ*AfcpcB* and C’*AfcpcB* conidia. MH-S murine alveolar macrophages were challenged with three-fold WT and Δ*afcpcB* conidia. Cultures were then incubated for 4 h at 37 °C in an atmosphere of 5% CO_2_. (**A**) Differential interference contrast microscopic views of adherent macrophages containing conidia. External conidia (black arrows) were stained by CFW (1 mg/mL in DPBS). White arrows indicate conidia endocytosed by macrophage cells. BF, bright-field microscopy; CFW, calcofluor white. (**B**) Phagocytosis indicates percentage of macrophages containing one or more ingested conidia (*n* = 21). Statistical analysis was performed using Student’s *t*-test. *** *p* < 0.001. (**C**) Phagocytosis index represents the average number of indigested conidia per macrophage (c/m) (*n* = 70). Statistical analysis was performed using Student’s *t*-test. ** *p* < 0.01.

To investigate the interaction between conidia and murine alveolar macrophages, adherent macrophages were challenged with conidia and co-cultured for 4 h. A significant decrease in the number of macrophages that ingested Δ*AfcpcB* conidia (36.1 ± 2.0%), compared to WT conidia (57.8 ± 1.9%) and C’*AfcpcB* conidia (56.8 ± 2.2%), was observed (Figure 4A,B). The phagocytic index, which represents the average number of conidia per macrophage, was also lower in macrophages containing Δ*AfcpcB* conidia (1.4 vs. 2.0 c/m) (Figure 4C). These in vitro data suggest that *AfcpcB*-deletion influences phagocytosis of conidia by possible changes in the cell wall components of the conidia surface.

### 3.5. The AfcpcB Is Required for Biosynthesis of Cell Wall Components and Hydrophobin

**Figure 5:**
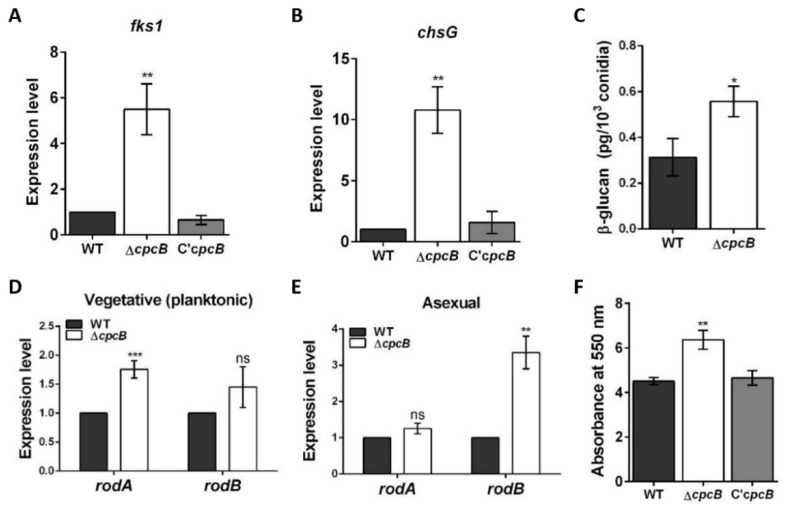
Role of *Af*CpcB in biosynthesis of cell wall components. Expression pattern of the (**A**) *fks1* and (**B**) *chsG* genes during vegetative growth (*n* = 4–8). Mycelia were cultured in liquid broth for 16 h. The RT-qPCR expression ratios were normalized using the 18S rRNA gene. (**C**) Amount of β-glucan (pg) per 10^3^ conidia in WT and Δ*AfcpcB* strains. (**D**) Expression pattern of the *rod* genes during vegetative growth. Mycelia were cultured in liquid broth for 16 h. (**E**) Expression patterns of the *rod* genes during asexual development. Mycelia were transferred onto GMM agar and incubated for 48 h. The RT-qPCR expression ratios were normalized using the 18S rRNA gene. (**F**) Biofilm formation. The biofilms were stained with 0.01% crystal violet and dissolved in 30% acetic acid solution. The amounts of dye were measured by spectrophotometry at 550 nm. Statistical analysis was performed using Student’s *t*-test. * *p* < 0.05, ** *p* < 0.01, *** *p* < 0.001. “ns” indicates not significant.

To determine why the Δ*AfcpcB* conidia can deter phagocytosis by alveolar macrophages, we investigated the expression of cell wall biosynthesis genes. The *fks1* and *chsG* genes encode β-1,3-glucan synthase and chitin synthase, respectively [52]. The *fks1* and *chsG* mRNA were upregulated in the Δ*AfcpcB* strain (Figure 5A,B). In accordance with the *fks1* expression pattern, the amount of β-1,3-glucan content was higher than that of WT (Figure 5C). These data suggested that *Af*CpcB is involved in the *A. fumigatus* cell wall biosynthesis.

Next, the expression of hydrophobin genes *rodA* and *rodB* was evaluated. Conidia are covered by outer layers, composed of the hydrophobins and melanin layers [17]. These layers protect conidia against host defense by masking the polysaccharides on the conidial surface, such as glucan and chitin, and hindering PRR-dependent recognition. We hypothesized that a change in hydrophobin composition in the Δ*AfcpcB* strain affects the PAMP–PRR interaction. The Δ*AfcpcB* strain showed a 1.8-fold increase in expression of the *rodA* gene during the vegetative stage (Figure 5D) and a 3.4-fold increase in the expression of the *rodB* gene during the asexual stage (Figure 5E). The extracellular matrix (ECM) of fungi is involved in biofilm formation [53]. In *A. fumigatus* biofilm condition, *rodA* and *rodB* were highly expressed [54]. To evaluate the ability of each strain to form biofilms on a solid surface, biofilms grown in 12-well plates were quantified using the crystal violet assay. Biofilm formation was increased in the Δ*AfcpcB* strain compared to WT and C’*AfcpcB* strain (Figure 5F). These results suggested that the *AfcpcB* strain causes a possible change in both inner and outer cell wall layers, thus affecting the capacity of PRRs in host immune cells to react with the *A. fumigatus* conidia surface.

### 3.6. AfCpcB Functions in Response to Cell Wall and Oxidative Stress

**Figure 6:**
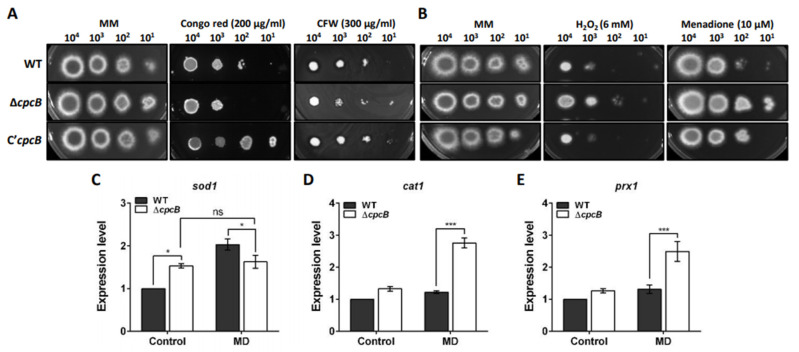
Cell wall and oxidative stress response mediated by *Af*CpcB. Conidia with 10-fold serial dilutions were spotted onto media and cultured at 37 °C for 2 days. (**A**) Sensitivity of the strains to cell wall stresses (200 μg/mL of Congo red and 300 μg/mL of CFW). CFW, Calcofluor-white. (**B**) Sensitivity of the strains to oxidative stresses (6 mM H_2_O_2_ and 10 μM MD). MD, menadione. Expression patterns of genes associated with oxidative stress response, (**C**) *sod1*, (**D**) *cat1*, and (**E**) *prx1* under MD treatment conditions (*n* = 4–8). Mycelia were cultured in liquid broth, transferred onto GMM (Control) and GMM containing 10 μM MD, and incubated for 6 hr. The RT-qPCR expression ratios were normalized using 18S rRNA gene. Statistical analysis was performed using Student’s *t*-test. * *p* < 0.05, *** *p* < 0.001. “ns” indicates not significantly different.

To elucidate the cellular functions of CpcB in *A. fumigatus*, the effect of *AfcpcB* deletion on the response to external stress was investigated. Understanding cell wall integrity signaling is important for the study of drug response and virulence. Many fungal mutants with defective cell walls are sensitive to antifungal reagents, such as CFW and CR [55,56]. CFW binds to β-1,3 and β-1,4 polysaccharides in chitin [57]. The fungus responds to CR, which is considered to bind to polysaccharides and form covalent links between chitin chains and β-glucan, by modifying its cell wall composition and permeability [58]. The Δ*AfcpcB* strain was sensitive to CR compared to WT (Figure 6A). When the response to osmotic stress (NaCl and sorbitol) and DNA replicative stress (hydroxyurea, methyl methanesulfonate, and benomyl) was examined, no significant abnormalities were observed in the Δ*AfcpcB* strain (Supplementary Figure S1A,B).

The capacity of scavenging ROS produced by host phagocytes is regarded as a putative virulence factor in pathogenic fungi [59]. While ROS are byproducts of normal aerobic metabolism that participate in the signaling pathway [60], ROS generated by host defense mechanisms can kill conidia and induce hyphal defects in *A. fumigatus* [61]. To characterize the role of *Af*CpcB in response to oxidative stress, we investigated the resistance of the Δ*AfcpcB* strain to H_2_O_2_ and MD. The Δ*AfcpcB* strain showed increased resistance to H_2_O_2_ and MD compared to WT (Figure 6B). We investigated the mRNA expression of several genes known to be involved in the oxidative response in *A. fumigatus*, and particularly in the presence of MD (10 μM): *sod1* encoding a copper-dependent superoxide dismutase (SOD), *cat1* encoding a mycelial catalase, and *prx1* encoding Cys-based peroxidase [62,63,64]. Upon MD treatment, the WT strain showed increased expression of *sod1* (about 2-fold); however, the Δ*AfcpcB* strain showed no remarkable change in expression patterns and continuous expression of *sod1* (Figure 6C). The expression levels of *cat1* and *prx1* were increased in the Δ*AfcpcB* strain in the presence of MD, compared to that in the WT strain (approximately 1.9-fold and 2.1-fold, respectively) (Figure 6D,E). Taken together, these results indicate that *Af*CpcB contributes to the transcriptional regulation of ROS scavenging enzymes, which are encoded by *cat1* and *prx1*, and upregulation of these genes in the Δ*AfcpcB* strain may contribute to resistance against oxidative stress.

### 3.7. The ΔAfcpcB Strain under Oxidative Stress Showed Increased Expression of Ergosterol Biosynthesis-Related Genes

**Figure 7:**
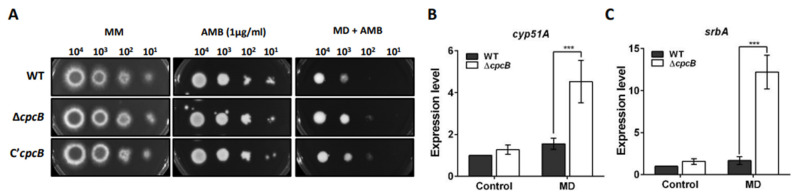
Resistance to AMB and expression pattern of *cyp51A* and *srbA* genes under oxidative stress conditions. (**A**) Sensitivity of the WT, Δ*AfcpcB*, and C’*AfcpcB* strains to 1 μg/mL of AMB in the presence and absence of 10 μM MD. Conidia with 10-fold serial dilutions were spotted onto media and the plates were then cultured at 37°C for 2–3 days. Expression pattern of genes associated with ergosterol biosynthesis, (**B**) *cyp51A*, and (**C**) *srbA* (*n* = 4). Mycelia were cultured in liquid broth, transferred onto GMM (Control) and GMM containing 10 μM MD (MD), and incubated for 6 h. The RT-qPCR expression ratios were normalized using the 18S rRNA gene. Statistical analysis was performed using Student’s *t*-test. *** *p* < 0.001. AMB, amphotericin B; MD, menadione.

Azoles inhibit fungal sterol 14-α demethylase (Cyp51/Erg11A), which is required for ergosterol biosynthesis [65]. Polyenes, such as AMB and nystatin, bind to ergosterol, which kills cells by allowing membrane permeabilization and extracting ergosterol in the membrane [66]. Cai et al. revealed that the Δ*AfcpcB* strain in a *veA1* and Δ*ku80* background—which is not a suitable model for sexual development—demonstrated increased resistance against antifungal drugs, including voriconazole, bifonazole, and AMB [9]. To verify whether the supermater Δ*AfcpcB* strain exhibited resistance against antifungal drugs, conidia were serially diluted and spotted onto GMM with AMB. The Δ*AfcpcB* strain exhibited no significant change in resistance against AMB (Figure 7A, AMB). To examine the function of CpcB in ergosterol biosynthesis, the expression of *cyp51A* and *srbA* encoding sterol regulatory element-binding protein (SREBP) was analyzed using RT-qPCR. SrbA is a transcription factor containing a basic helix-loop-helix (bHLH) leucine zipper DNA binding domain and is required for cell polarity, hypoxia response, and azole drug resistance [25]. When overnight-cultured mycelia were transferred onto GMM agar, the expression pattern of both *cyp51A* and *srbA* genes showed no remarkable change between the WT and Δ*AfcpcB* strains (Figure 7B,C, control). To adapt to oxidative stress caused by MD, ergosterol biosynthesis is repressed in yeast [67]. We next investigated whether oxidative stress would cause a transcriptional adaptation of ergosterol biosynthesis. The Δ*AfcpcB* strain exhibited slightly increased resistance when MD was added to the AMB-containing medium (Figure 7A, MD + AMB). Under oxidative stress, the Δ*AfcpcB* strain showed increased expression of both the *cyp51A* and *srbA* genes (approximately 2.9-fold and 7.3-fold, respectively) (Figure 7B,C, MD). These data suggested that deletion of the *AfcpcB* gene causes dysregulation of the *cyp51A* and *srbA* expression under oxidative stress conditions.

## 4. Discussion

Although CpcB was found to be required for vegetative growth, germination, asexual development, and gliotoxin production in *A. fumigatus* [8] and growth and development of *A. nidulans* [11], the *cpcB* deletion in the *A. fumigatus* supermater strain showed no significant change in vegetative growth and asexual development (data not shown). Further investigation is required to explain the strain-dependent variation, however, it is noteworthy that a number of studies demonstrate significant interstrain variability with respect to phonotypes, immune responses, and virulence in various models of infection [68].

While mature cleistothecia of *A. nidulans* are black spheres with numerous red-purple ascospores [37,69], the Δ*AncpcB* strain produces small immature bright-red cleistothecia with no ascospores and a reduced number of cleistothecia and ascospores [11]. Based on the fact that *An*CpcB is important in sexual development in *A. nidulans*, we investigated whether *Af*CpcB plays a crucial role in the sexual development in *A. fumigatus*. To investigate the role of *Af*CpcB in sexual development, the use of the supermater strain as a recipient for the generation of deletion-strain was indispensable. Therefore, we constructed an *AfcpcB*-deletion mutant using the supermater strain AFB62 as a recipient strain and crossed it with the supermater strain AFIR928 with the opposite mating type. The Δ*AfcpcB* × AFIR928 cross did not affect the size of cleistothecia, but rather the production of cleistothecia and the formation of ascospores (Figure 2). This is the first report to demonstrate that *Af*CpcB is involved in sexual development in *A. fumigatus*.

Unlike sexual induction in *A. nidulans*, the VeM method in *A. fumigatus* on oatmeal agar produced both asexual (conidia) and sexual (cleistothecia) reproductive organs [14]. Given the difficulties of molecular analysis using sexual developmental cultures in *A. fumigatus*, we investigated transcription patterns in cleistothecia that contain many asci and ascospores. The Δ*AfcpcB* × AFIR928 cross showed decreased *veA*, *steA*, and *vosA* transcription (Figure 3). Studies using Δ*AncpcB* and Δ*AnsvfA* strains have previously shown that proper expression of the *veA* gene is required for sexual development in *A. nidulans* [11,37]. These data suggest that CpcB is involved in the regulation of *veA* gene expression during sexual development in *A. nidulans* and *A. fumigatus*. In some filamentous fungi including *A. nidulans*, *STE12* mutants showed impaired sexual reproduction [50,70,71,72]. The absence of cleistothecial primordia or foci indicates that SteA is required for an early sexual cycle. The *vosA* gene is expressed in conidia and ascospores for viability in *A. nidulans* [51]. Immature cleistothecia that contain no ascospores from the Δ*AfcpcB* × AFIR928 cross showed significantly reduced *vosA* expression, suggesting that VosA is expressed in ascospores in *A. fumigatus*. Although transcription patterns were not investigated at different developmental stages, these data indicate that *Af*CpcB is required for cleistothecia maturation by controlling the expression of genes such as *steA* and *vosA*.

Fungi engage in transcriptional, post-translational, and enzymatic strategies against oxidative stress, and such strategies include SOD, catalase, and peroxidase activities [73]. The Δ*AfcpcB* strain showed increased resistance to both H_2_O_2_ and MD (Figure 6B). MD generates intracellular ROS through the accumulation of superoxide anions and mitochondrial depolarization, which induces apoptosis [73,74,75]. As superoxide anions are unstable, they are converted to H_2_O_2_ either spontaneously or by SOD. Moreover, while the WT strain responds to MD treatment by inducing *sod1* expression, the Δ*AfcpcB* strain continuously expresses the *sod1* gene both under normal and oxidative stress conditions (Figure 6C). H_2_O_2_ is detoxified by various ROS scavenging enzymes, such as catalase, glutathione peroxidase, and thioredoxins. Our data showed upregulated expression of *cat1* and *prx1* genes in the Δ*AfcpcB* strain against MD treatment (Figure 6D,E), which can explain the increased resistance against oxidative stress in the spotting assay. These data suggest that CpcB negatively regulates the expression of *cat1* and *prx1* genes under oxidative stress conditions.

Oxidative stress represses the transcription of the ergosterol biosynthesis gene (ERG) and causes decreased levels of cellular ergosterol in the budding yeast, *Saccharomyces cerevisiae* [67]. In the fission yeast *Schizosaccharomyces pombe*, ergosterol levels are repressed during oxidative stress [76]. Transcriptomic and proteomic analyses revealed that hypoxia affects metabolic changes, such as ergosterol biosynthesis, in *A. fumigatus* [77]. A Zn_2_-Cys_6_ transcription factor AtrR positively regulates sterol biosynthesis [78]. The CCAAT-binding domain complex (CBC), a heterotrimer comprising HapB, HapC, and HapE, is a negative regulator of sterol biosynthesis [79]. Binding of the CBC at the *cyp51A* promoter is facilitated by another transcriptional regulator, an iron-responsive bZIP transcription factor HapX. SrbA directly regulates ergosterol transcription during hypoxia [80]. These studies suggest that regulation of ergosterol biosynthesis is required for an adaptive response to extracellular or intracellular stimuli, including ROS. Understanding the *Aspergillus* ergosterol biosynthesis pathway is also important to facilitate the development of antifungal drugs [81]. The Δ*AfcpcB* strain, generated in this study using the supermater AFB62, exhibited enhanced resistance in GMM containing both MD and AMB (Figure 7A). While WT showed no remarkable change in *srbA* and *cyp51A* gene expression under oxidative stress conditions, the Δ*AfcpcB* strain induced *cyp51A* and *srbA* expression under oxidative stress conditions (Figure 7B,C). These data indicate that, unlike yeast, transcriptional repression of ERG is not always necessary under MD-treated conditions in *A. fumigatus*. There is limited information on the mechanisms for ergosterol biosynthesis under oxidative stress conditions in *A. fumigatus*; however, our results suggest that *Af*CpcB plays a role in the negative regulation of *cyp51A* and *srbA* gene expression under oxidative stress conditions.

In this study, we report the involvement of *Af*CpcB in phagocytosis by alveolar macrophages for the first time. When we tested phagocytosis of murine alveolar macrophages challenged with conidia, the phagocytosis percentage and indigested number of Δ*AfcpcB* conidia by macrophages as values were reduced (Figure 4). These data suggest that *Af*CpcB affects the mechanisms of fungal recognition and internalization by macrophages. The inner cell wall components of conidia, such as glucan, galactomannan, and chitin, are recognized by PRRs in host cells [15,61]. The Δ*AfcpcB* strain decreased the resistance of *A. fumigatus* to CR (Figure 6A), and increased expression of cell wall genes and amounts of β-glucan (Figure 5), suggesting that *Af*CpcB modifies the properties of the cell wall surface. Cai et al. showed that the electron-dense pigment layer of the cell wall surface in both *cpcB* and *gpaB* (encoding Gα subunit)-deletion strains showed disrupted phenotypes in cell wall integrity in *A. fumigatus* [8]. In addition, the fungal cell wall features an outer layer composed of hydrophobins (rodlet proteins) and melanin, which mask cell wall PAMPs in conidia [82]. Among the seven rodlet proteins (RodA–RodG) in *A. fumigatus*, RodA is required for the formation of a rodlet layer and protection from phagocytosis by alveolar macrophages [21]. The *rodA* gene is not differentially expressed in planktonic culture but highly expressed in sporulating culture [54]. Although RodB is not required for rodlet formation and Δ*rodB* did not show any phenotype in biofilm conditions, the *rodB* gene is highly expressed in biofilm and in mice invasive aspergillosis model [21,54,82]. While the *rodB* gene is not differentially expressed in asexual culture, the Δ*AfcpcB* showed an increase in *rodA* and *rodB* genes in planktonic and asexual culture, respectively (Figure 5D,E), suggesting that *Af*CpcB is involved in the regulation of expression of hydrophobin genes. Taken together, these results suggest that *Af*CpcB might affect the phagocytosis by alveolar macrophages by regulating cell wall integrity and the formation of hydrophobins.

Interestingly, Cai et al. showed that the virulence of the Δ*AfcpcB* strain in a *veA*1 and Δ*ku80* background is attenuated in an immunosuppressed mouse model (survival rate of 60%) [8]. While the Δ*AfcpcB* strain in a *veA1* and Δ*ku80* background showed a decreased growth rate, defects in asexual reproductive organs, and increased resistance against antifungal drugs, our Δ*AfcpcB* strain showed a similar growth rate with WT [8], defects in sexual reproductive organs, and increased resistance against oxidative stress. It is necessary to confirm the virulence of our Δ*AfcpcB* strain using an in vivo model system.

To summarize, we found that *Af*CpcB plays important roles in sexual development and the phagocytosis by alveolar macrophages. Based on our data together with the results of previous studies, we conclude that CpcB, a Gβ-like protein, might have functions that are involved in oxidative stress response and maintenance of cell wall properties.

## Data Availability

Not applicable.

## Supplementary Materials

The following supporting information can be downloaded at: https://www.mdpi.com/article/10.3390/jof8010056/s1, Figure S1: Effects of *AfcpcB*disruption on sensitivity to osmotic and DNA replicative stresses. Conidia with 10-fold serial dilutions were spotted onto media and then, the plates were cultured at 37 °C for 2 days. (A) Sensitivity of the strains to osmotic stresses (1.5 M NaCland 2.5 M sorbitol). (B) Sensitivity of the strains to DNA replicative stresses (15 mM HU, 10 μMMMS and 0.6 μg/mL of benomyl). HU, hydroxyurea; MMS, methyl methanesulfonate. Table S1. Primers used in this study.
