# Supplementary material for: The Gβ-like Protein AfCpcB Affects Sexual Development, Response to Oxidative Stress and Phagocytosis by Alveolar Macrophages in Aspergillus fumigatus
Source: J Fungi (Basel). 2022 Jan 6;8(1):56. doi: 10.3390/jof8010056 (PMC8777951; doi:10.3390/jof8010056)
Supplement: Supplementary file 1 [file jof-08-00056-s001.zip › jof-1529278-SI/Figure S1.pdf]

Supplementary information

The Gβ-like Protein *Af*CpcB is Required for Sexual Development, Response to Oxidative Stress and Phagocytosis by Alveolar Macrophages in *Aspergillus fumigatus*

Joo-Yeon Lim, Yeon-Ju Kim and Hee-Moon Park*

Department of Microbiology and Molecular Biology,

College of Bioscience and Biotechnology,

Chungnam National University, Daejeon 34134, Korea

* To whom correspondence should be addressed.

Hee-Moon Park, Tel: 042-821-7553/ Fax : 042-822-7367, E-mail: hmpark@cnu.ac.kr

Supplementary Figure S1.

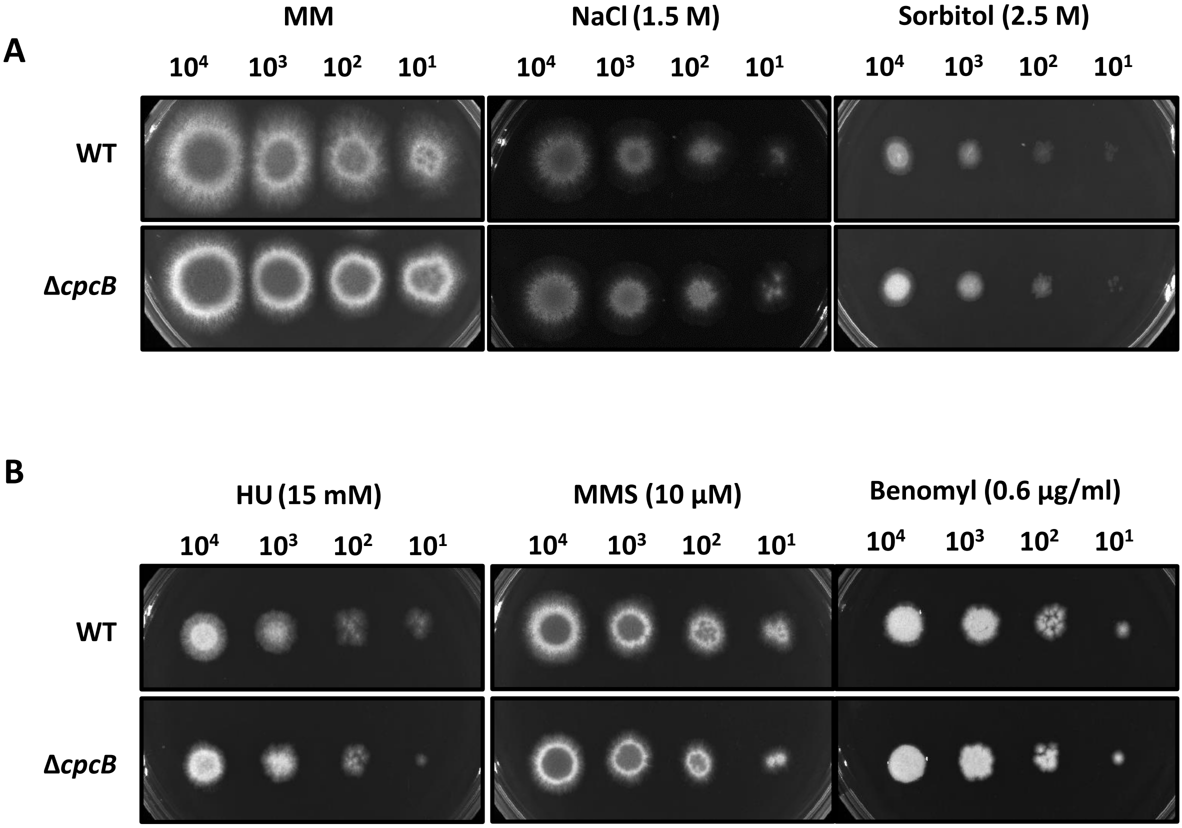

Supplementary Figure S1. Effects of *AfcpcB* disruption on sensitivity to osmotic and DNA replicative stresses. Conidia with 10-fold serial dilutions were spotted onto media and then, the plates were cultured at 37°C for 2 days. (A) Sensitivity of the strains to osmotic stresses (1.5 M NaCl and 2.5 M sorbitol). (B) Sensitivity of the strains to DNA replicative stresses (15 mM HU, 10 μM MMS and 0.6 μg/ml of benomyl). HU, hydroxyurea; MMS, methyl methanesulfonate.
